# Regional differences in the incidence of Alzheimer’s disease and related dementias in South Carolina

**DOI:** 10.3389/fneur.2025.1584127

**Published:** 2025-09-05

**Authors:** Daniel A. Amoatika, Maggi C. Miller, Robert J. Adams, Nicholas J. Milano

**Affiliations:** ^1^Department of Epidemiology and Biostatistics, University of South Carolina, Columbia, SC, United States; ^2^Office for the Study of Aging, University of South Carolina, Columbia, SC, United States; ^3^Department of Neurology, Medical University of South Carolina, Charleston, SC, United States

**Keywords:** Alzheimer’s disease, dementia, incidence, public health regions, South Carolina

## Abstract

**Introduction:**

There is an increase in the population of older adults, 65 years or older in South Carolina. Socio economic and environmental factors are linked to the diagnosis of Alzheimer’s Disease and related dementias (ADRD). The aim of this study was therefore, to characterize the incidence of ADRD by public health regions (PHR).

**Methods:**

We estimated the incidence of ADRD for 2021using data from South Carolina Alzheimer’s Disease Registry (SCADR) and the Annual County Resident Population Estimates (ACRPE). The incidence of new cases per 100, 0000 population for each county, and PHR, and age-adjusted ADRD specific diagnosis were estimated. Poisson regression modeling was used to compare crude and ADRD specific incidence by PHRs. The incidence of ADRD by counties and PHRs was visualized using TIGERline files.

**Results:**

A total of 18,955 registrants from the SCADR were included in this study. About 38% of the participants were between 75 and 84 years. Additionally, about 79% of the registrants had Alzheimer’s (AD) diagnosis. The crude incidence of ADRD was higher in the Pee Dee PHR (896 per 100,000). Among ADRD specific diagnosis, AD incidence was higher in the Pee Dee PHR (727 per 100,000), Vascular dementia (VaD) and Mixed dementia incidences were higher in the Upstate PHR. The crude incidence of ADRD differed significantly across all the PHRs (*p* < 0.05).

**Discussion:**

Regional differences in the incidence of ADRD suggest possible disparities in healthcare access, socioeconomic conditions and geographical factors. Targeted interventions, and early screening among young populations should be prioritized.

## Introduction

Dementia is a clinical condition resulting from progressive neurodegeneration and characterized by impairment in two or more cognitive domains such as memory, language, executive functioning and attention that significantly interfere with activities of daily functioning. Progressive damage to brain cells caused by underlying conditions that lead to structural and functional changes in the brain (1, 2). Although Alzheimer’s disease (AD) is the most common type of dementia, other related dementias include vascular dementia (VaD), Mixed dementia, dementia with Lewy bodies, and frontotemporal dementia (3). Alzheimer’s disease and related dementias (ADRD) mostly affect older adults in the population, and data suggest that its effect on individuals, caregivers, and society at large will continue to increase as the older population ages (4). Additionally, there is currently no cure for ADRD, although preventive measures including healthy lifestyle-factors have decreased the age-specific incidence in high income countries. However, a significant increase in life expectancy suggests that the number of people living with ADRD will continue to rise (5).

Globally, a new case of dementia is diagnosed/developed every 3 s, and in the United States (US), a new case of dementia is diagnosed every 65 s (6). ADRD is underdiagnosed in America, with data suggesting that a large proportion of Americans do not know they have it (7). Underdiagnosis of ADRD is most common among minority older adults (8, 9).

The population of South Carolina is aging. The population of older adults 65 years or older increased from about 14 to 19% in 2021, with one in five residents being 65 years or older (10). Due to this, ADRD is a growing problem in South Carolina. It has been suggested that where one resides may influence one’s likelihood of being diagnosed with ADRD (11). Additionally, research has shown a higher likelihood of underdiagnosis among minority populations, attributed to stigmatization and generally low care-seeking behavior (12).

South Carolina is divided into four public health regions (PHR): Low Country, Midlands, Pee Dee, and Upstate, each characterized by distinct social and cultural attributes that impact the well-being of its inhabitants. Health outcomes, population growth, educational attainment, and other factors that influence the health of older adults vary in these regions. The Pee Dee PHR has the highest social vulnerability index, which is determined by factors including poverty, and lack of access to transportation and housing community (10). This may make counties in the Pee Dee more vulnerable to public health events. Previous research has also shown that high social vulnerability index can lead to inequitable access to health care and essential services (13, 14).

The South Carolina Alzheimer’s Registry (SCADR) is the oldest and most comprehensive registry in the US. Estimating the incidence of ADRD by PHRs in South Carolina can therefore help in scaling surveillance efforts for ADRD. Also, estimating the incidence of ADRD can help detect regional differences and the possible connection with region-specific characteristics including healthcare access, socioeconomic conditions, and environmental factors. In South Carolina, heart disease and stroke are among the top 10 leading causes of death for older adults, with disproportionately higher impact on non-Hispanic Black individuals compared to non-Hispanic White individuals (11). There is a complex bidirectional relationship between heart disease, stroke, and VaD, both heart disease and stroke may be risk factors for VaD (15). In addition, risk factors for heart disease and stroke, such as hypertension, are also risk factors for AD.

The aim of this study was therefore to characterize the incidence of ADRD by PHR and to determine if these incidences differ across the public health regions.

## Methods

### Data source

We used data from the 2021 South Carolina Alzheimer’s Disease Registry (SCADR) because it was the most recent and complete data available. The SCADR collects information on ADRD diagnosis and has been in existence since 1988. The SCADR collects information on individuals diagnosed with ADRD, including year of diagnosis, year of entry into the registry, location of diagnosis, current age of registrants, ADRD subtype, county of residence, zip code of registrants, date of birth, source of inclusion, month of death, year of death, underlying cause of death, sex of registrants, race, length of time being included in the registry, etc. Individuals with ADRD are identified/diagnosed by a physician/neurologist following a healthcare visit. A physician’s diagnosis is collected from the individual’s medical records through the diagnosed codes using the International Classification of Diseases, 10th revision Clinical Modification. People with ADRD diagnosis are added to the registry from various sources including South Carolina inpatient hospitalizations, longer-care Evaluations, Emergency departments, vital records, State health plans and other claims data (16). ADRD patients are classified into four categories in the Registry, Alzheimer’s disease, Vascular Dementia, Mixed Dementia and Other Medical Conditions (Alcohol dementia, Drug-induced dementia, Dementia with other conditions, Dementia with Lewy bodies, Pick’s Disease, Frontotemporal dementia). The registry core data set consists of case-identifying data for matching purposes, to remove duplicate records and for linking to other data sources.

**Figure 1:**
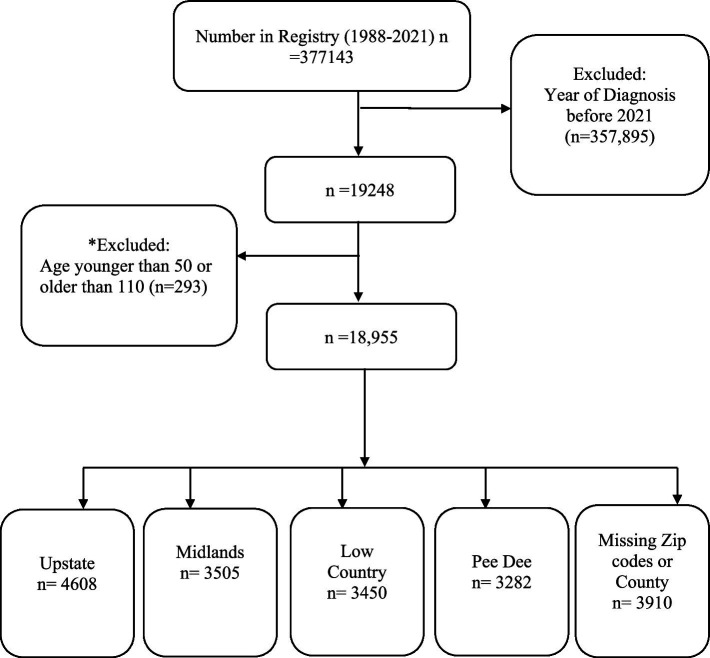
Analytical sample size. *293 participants outside the age range 50–110 years were excluded because of the possibility of data entry/measurement error misrepresenting the data.

In this study we estimated ADRD incidence per 100, 000 South Carolina population for 2021 using the SCADR 2021 Registry data (*N* = 18,955; Figure 1).

### Measures

#### Incidence

Age-adjusted incidence rates were calculated using the SCADR and the Annual County Resident Population Estimates (ACRPE). The ACRPE incorporate the 2020 census, Vintage 2020 estimates, and 2020 Demographic Analysis estimates (17).

Incidence was calculated as the number of new cases per 100,000 population, for each county, and PHR. Additionally, age-adjusted incidence for ADRD specific diagnosis were calculated using the 2020 US census as standard population.

#### Demographic characteristics

We included only participants in the Registry who are between 50 years (to capture early onset of ADRD) and 110 years. We grouped age as; less than 65 years, 65–74 years, 75–84 years, and 85 years and above.

Race was operationalized as non-Hispanic White individuals, non-Hispanic Black (African American) individuals or Other individuals. We combined Asian, American Indian, Other than listed race, Hispanic and unknown races as ‘Other individuals’ because of the small numbers of these in the Registry. Sex was male or female.

#### Statistical analysis

The age-adjusted incidence rates were calculated for each county and PHR using Microsoft Excel and SAS version 9.4 (SAS Institute, Cary, NC). Overall regional incidence rates were estimated, and incidence rates for ADRD specific diagnosis (Alzheimer’s Disease, Vascular Dementia, Mixed Dementia and Other) by PHRs and age-groups were also estimated.

Poisson regression with Tukey adjusted pairwise comparison was used to compare the crude incidence by PHRs and compare the ADRD specific incidence by PHRs to determine any significant differences in the incidence rates.

We modeled new cases (dependent variable) by PHR (independent variable), treating PHR as a fixed effect. The model also included the natural logarithm of the population at risk as an offset variable to account for rates (rather than raw counts) and to be able to compare PHR since they have different population sizes. A log-link function was applied to relate the expected number of new cases to the explanatory variable. The type3 option was included in the model to estimate the likelihood ratio (to assess the overall significance of the PHR effect).

The regression coefficients (ββ) from the model were exponentiated to derive incidence rate ratios (IRRs), allowing for comparison across PHRs and easy interpretability. We reported the *p*-values and confidence intervals (CIs) for the IRRs as a test of statistical significance (statistical significance set at *p* < 0.05).

QGIS version (3.34.13-Prizren) to visualize the incidence of ADRD by counties and ADRD specific diagnoses by PHRs using TIGERLine files (18).

#### Missing data

We assessed whether excluded missing data (missing county or zip codes) were associated with age, sex or specific ADRD diagnosis using chi-square test of independence. We evaluated the effect sizes using Cramer’s V with values of 0.10, 0.30 and 0.50 representing small, moderate and large associations between missing data and the specified variables, respectively. Sensitivity analyses were conducted using proportional redistribution method and extreme-case analysis.

## Results

### Sociodemographic characteristics

A total of 18,955 participants from the SCADR were included in this study. Most of the participants (38%) participants were between 75 and 84 years, and a few (8%) were less than 65 years. More than three-quarters (79%) of the study participants had Alzheimer’s disease (AD) diagnosis, and about 2% had Mixed dementia diagnosis. More than half (59%) of the study participants were females. White individuals represented about 66% of the study population and about 15% were other (Asian, American Indian, Hispanic; Table 1).

**Table 1:** The sociodemographic characteristics of the study participants in the SCADR.

Characteristic	Number of participants (%)				
	All participants	Upstate	Midlands	Pee Dee	Low country
ADRD					
Alzheimer’s Disease	14,896 (78.59)	3,590 (77.91)	2,844 (81.14)	2,801 (81.19)	2,827 (81.19)
Vascular	1,421 (7.50)	323 (7.01)	194 (5.53)	189 (5.48)	193 (5.54)
Mixed	442 (2.33)	133 (2.89)	56 (1.60)	80 (2.32)	63 (1.81)
Other	2,196 (11.59)	562 (12.20)	411 (11.73)	380 (11.01)	399 (11.46)
Age Group					
<65	1,342 (7.58)	259 (6.01)	187 (5.72)	167 (5.22)	207 (6.40)
65–74	3,403 (19.23)	753 (17.48)	555 (16.98)	543 (16.98)	677 (20.95)
75–84	6,755 (38.18)	1,685 (39.12)	1,284 (39.29)	1,234 (38.60)	1,262 (39.05)
≥85	6,194 (35.01)	1,610 (37.38)	1,242 (38.00)	1,253 (39.19)	1,086 (33.60)
Sex					
Male	7,220 (41.04)	1912 (41.49)	1,375 (39.23)	1,450 (42.04)	1,490 (42.79)
Female	10,374 (58.96)	2,696 (58.51)	2,130 (60.77)	1999 (57.96)	1992 (57.21)
Race					
White individuals	12,537 (66.14)	3,854 (83.64)	2,594 (74.01)	2,398 (69.51)	2,474 (71.05)
African American/Black individuals	3,574 (18.86)	623 (13.52)	828 (23.62)	920 (26.67)	921 (26.45)
Other individuals	2,844 (15.00)	131 (2.84)	83 (2.37)	132 (3.82)	87 (2.50)

The total number of participants across the four PHRs does not equal the overall number of participants due to missing regional classification for some cases.

In the PHRs, most incident AD cases were in the Midlands, Pee Dee, and Low Country. Most participants were between 74 and 84 years old in all the PHRs except for the Pee Dee, where the majority of their cases (39%) were 85 years or older. Additionally, participants less than 65 years old or aged between 65 and 74 years old were mostly from the Low Country PHR (60 and 21%, respectively; Table 1).

### ADRD incidence by PHR

**Figure 2:**
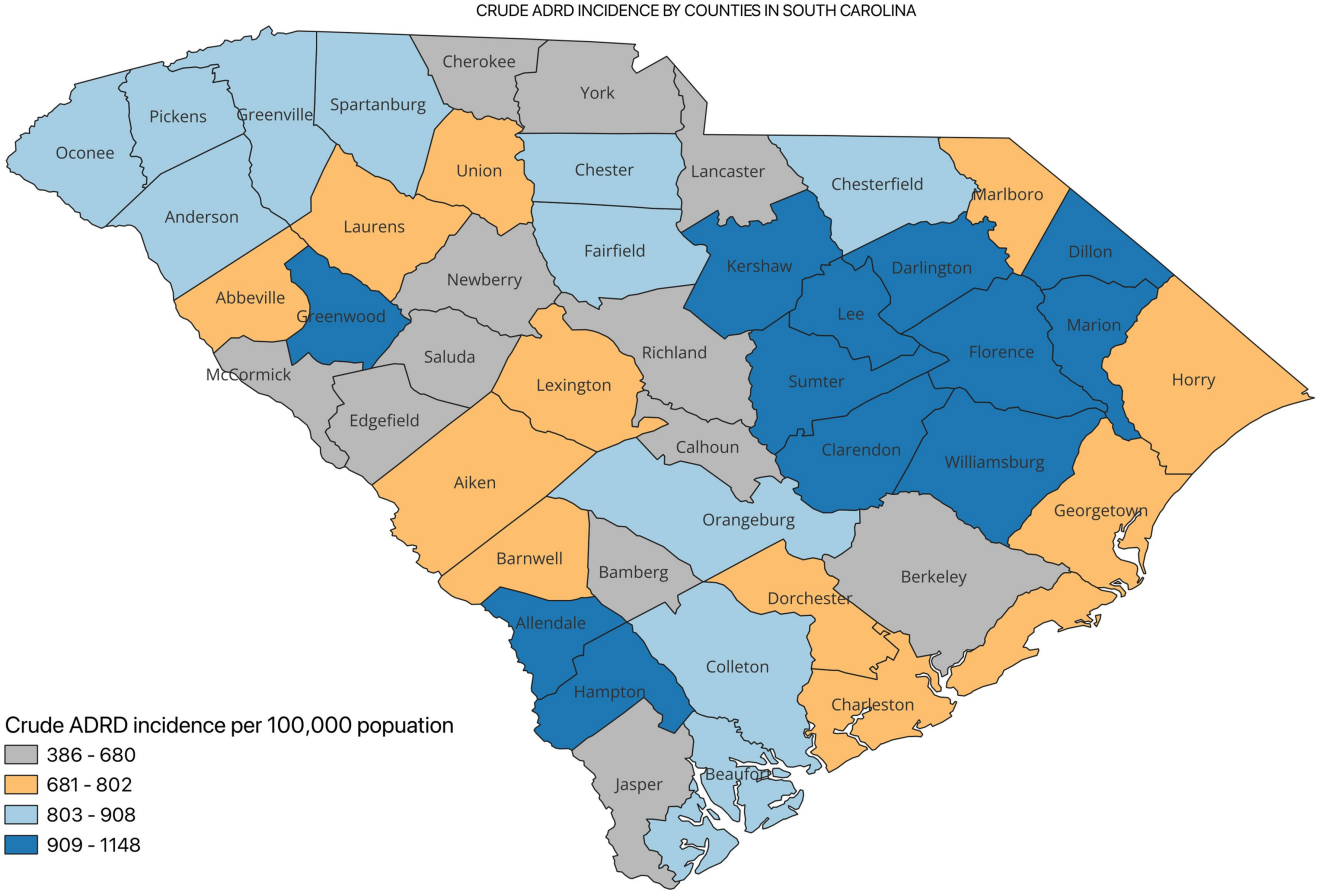
Crude ADRD incidence by counties in South Carolina.

**Figure 3:**
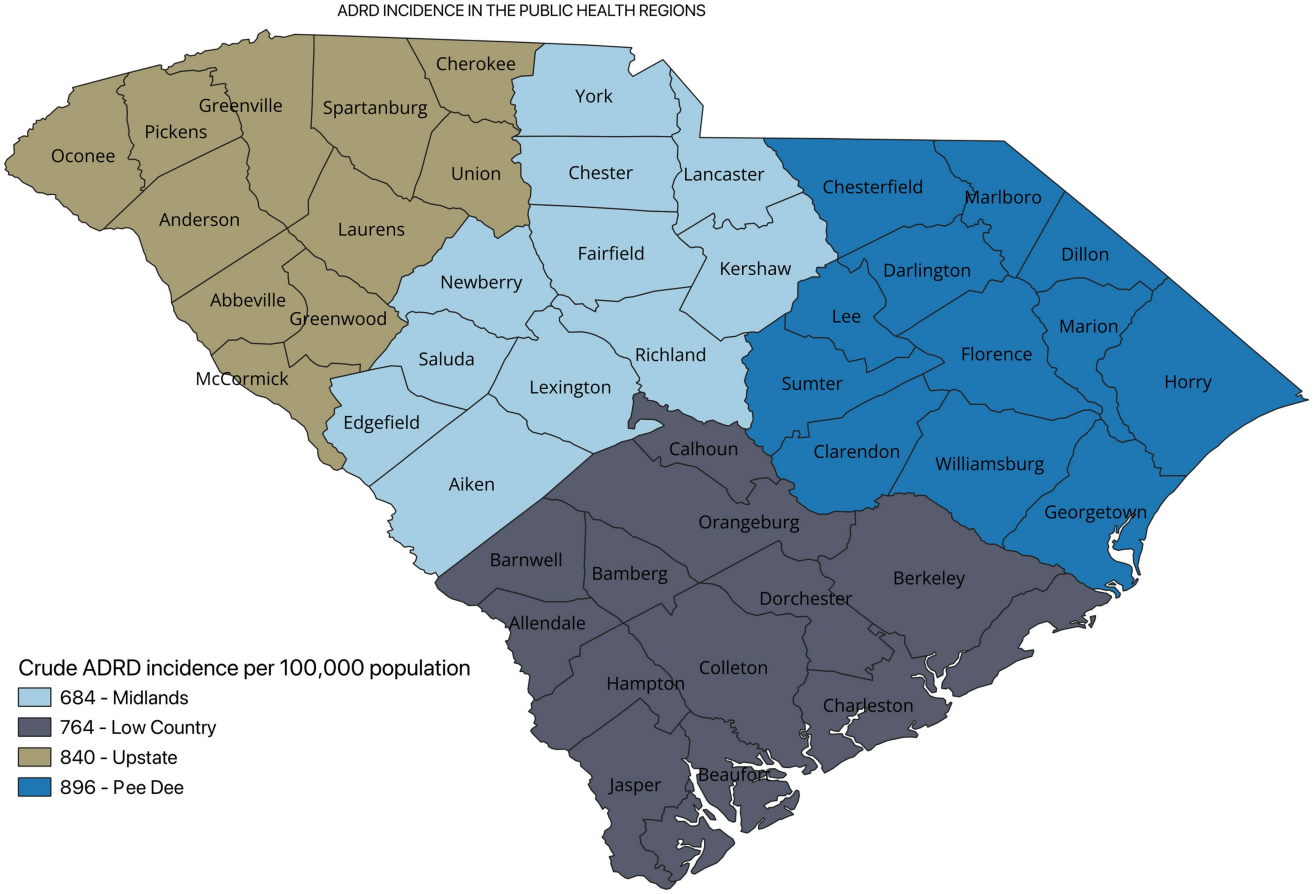
Crude ADRD incidence in the public health regions in South Carolina.

**Figure 4:**
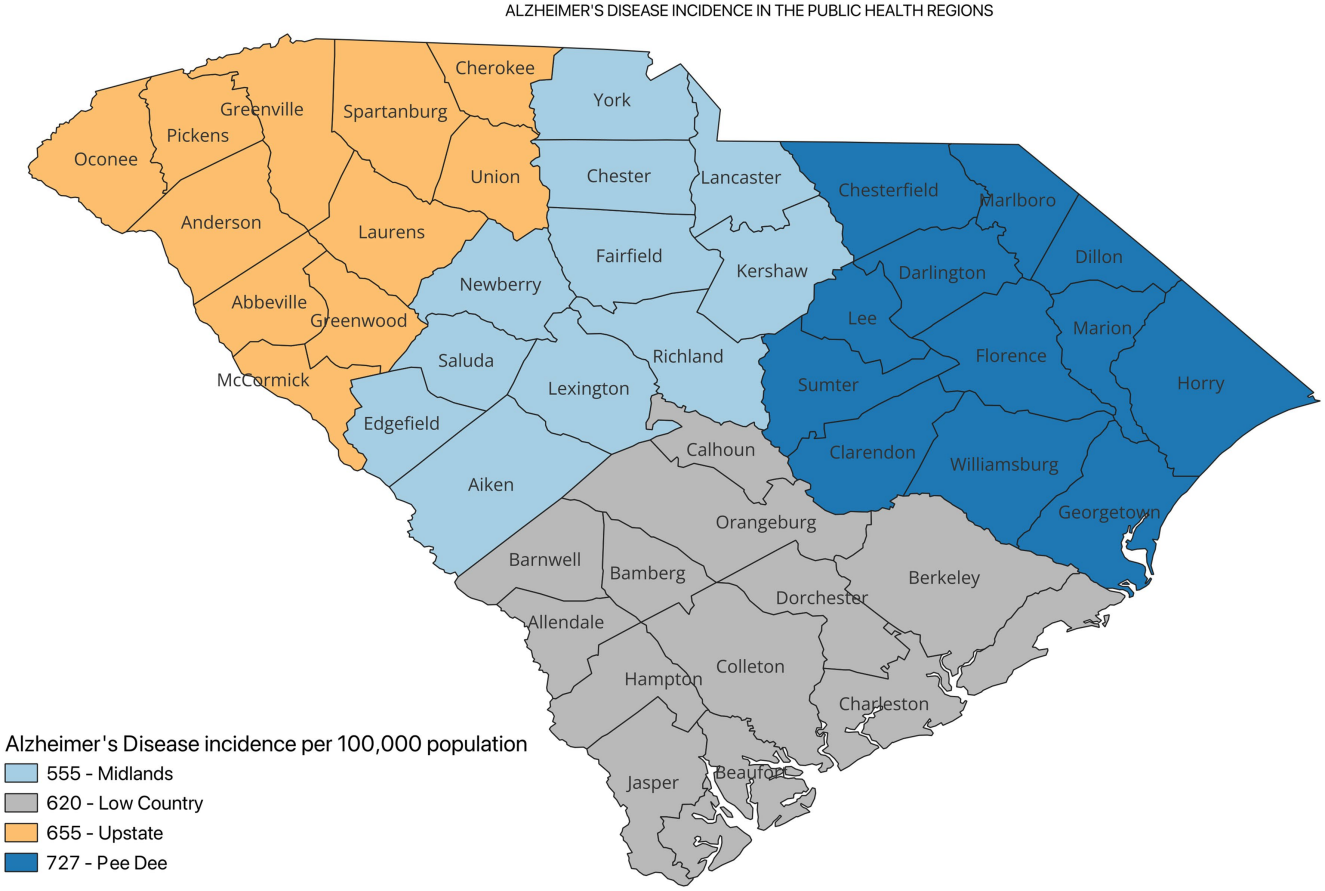
Alzheimer’s Disease incidence in the public health regions in South Carolina.

**Figure 5:**
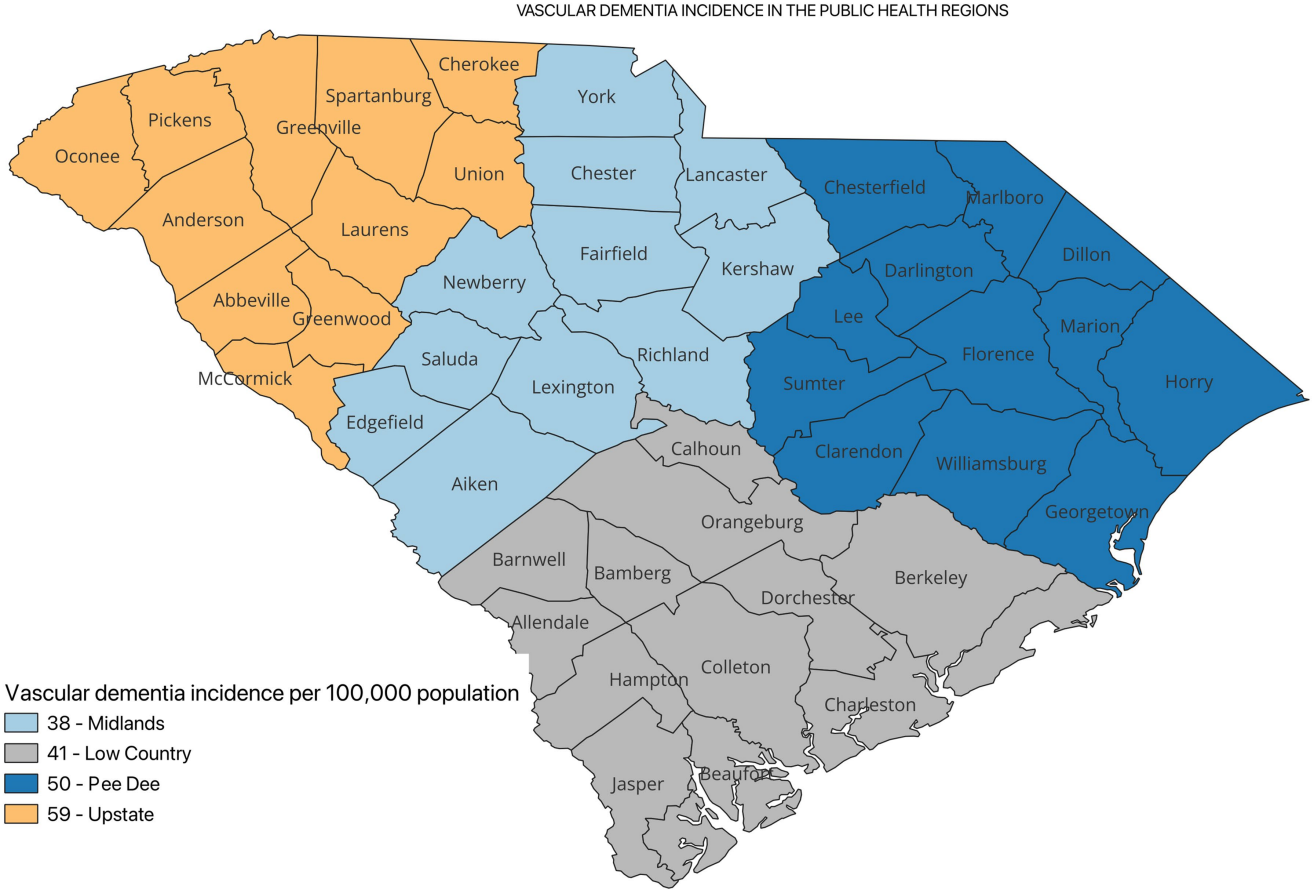
Vascular dementia incidence in the public health regions in South Carolina.

**Figure 6:**
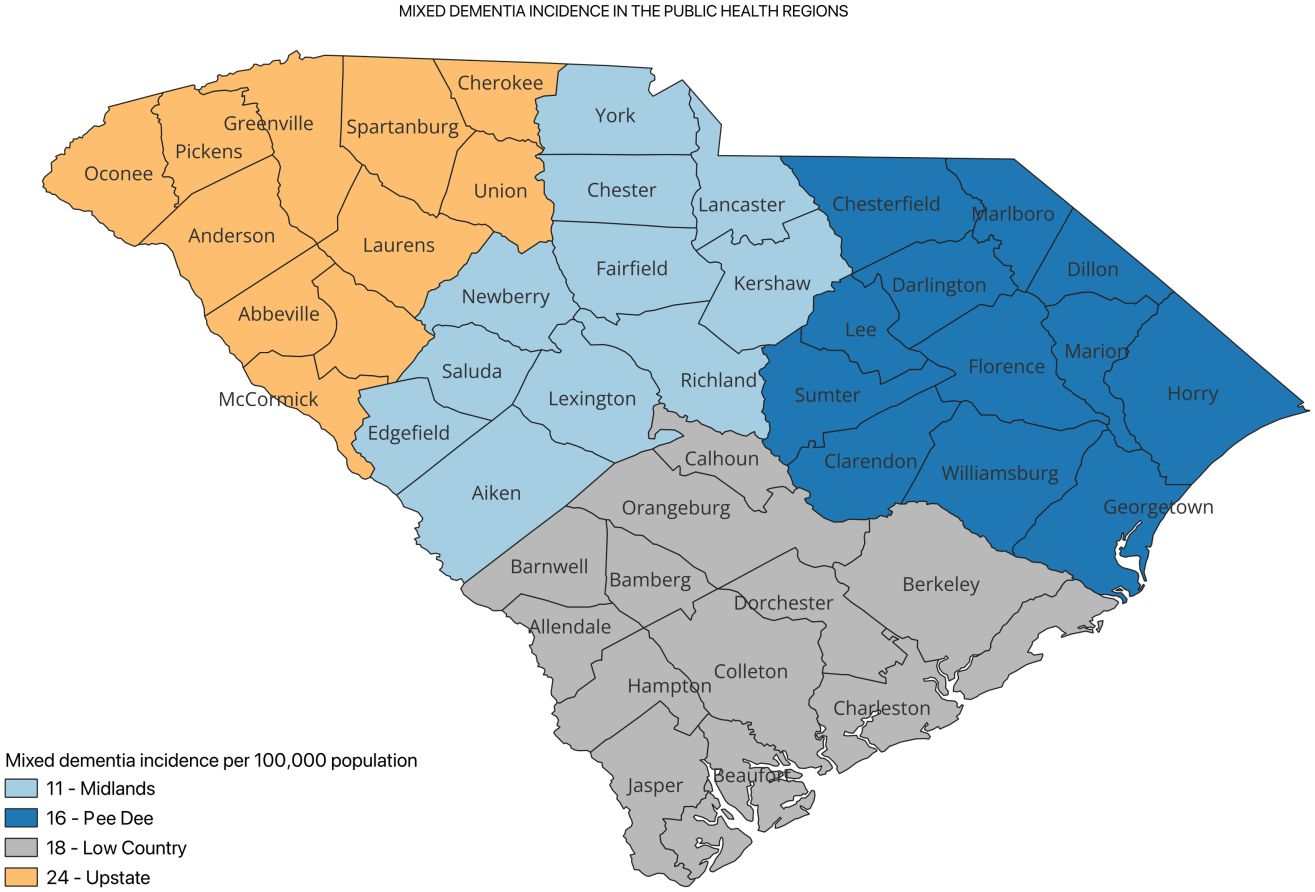
Mixed dementia incidence in the public health regions in South Carolina.

**Figure 7:**
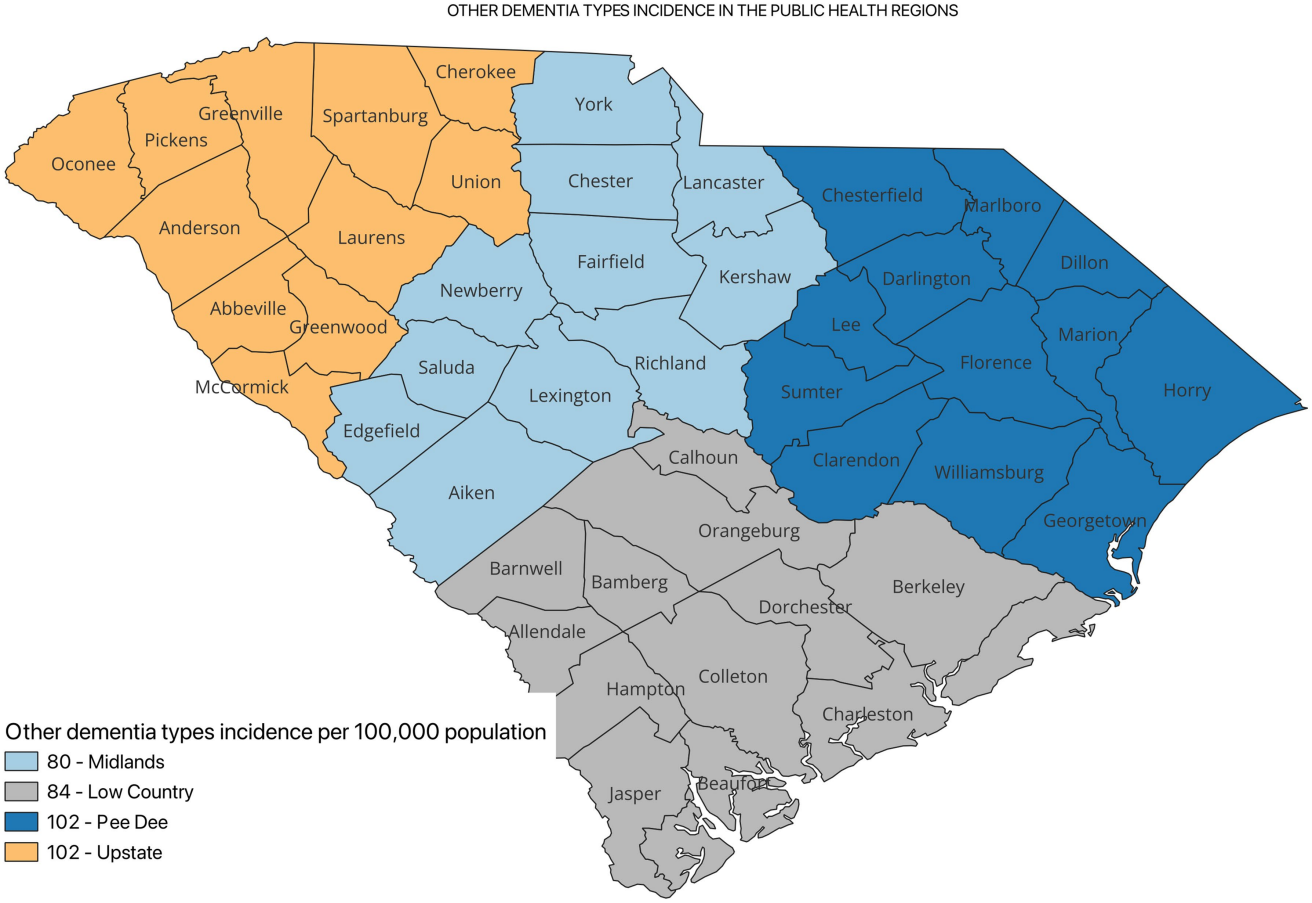
Other dementia types incidence in the public health regions in South Carolina.

Figure 2 shows the incidence of ADRD by county for 2021. Overall, the crude incidence was highest in the Pee Dee region (896 per 100,000 population) compared to other PHRs (Figure 3). Among ADRD-specific diagnoses, Alzheimer’s Disease (AD) incidence was also highest in Pee Dee (727 per 100,000; Figure 4). In contrast, the Upstate region recorded the highest incidence of Vascular dementia (59 per 100,000) and Mixed dementia (24 per 100,000; Figures 5, 6, respectively). Incidence of other dementia types was highest in both the Pee Dee and Upstate regions (102 per 100,000; Figure 7).

The crude incidence of ADRD differed statistically significantly across all the PHRs (*p* < 0.05; Table 2).

**Table 2:** Comparing crude incidence and ADRD specific incidence by PHR in the SCADR.

PHR	Crude incidence		AD		VaD		Mixed		Other	
	IRR	*p* value (95% CI)	IRR	*p* value (95% CI)	IRR	*p* value (95% CI)	IRR	*p* value (95% CI)	IRR	*p* value (95% CI)
Low Country vs. Midlands	1.118	**<0.0001 (1.066–1.171)**	1.118	**0.0002 (1.061–1.178)**	1.106	0.7571 (0.905–1.351)	1.622	**0.0282 (1.153–2.282)**	1.049	0.9040 (0.913–1.207)
Low Country vs. Pee Dee	0.852	**<0.0001 (0.814–0.894)**	0.853	**<0.0001 (0.809–0.899)**	0.843	0.3393 (0.689–1.030)	1.093	0.9524 (0.786–1.520)	0.823	0.0315 **(0.714–0.946)**
Low Country vs. Upstate	0.909	**0.0001 (0.869–0.950)**	0.947	0.1402 (0.902–0.995)	0.711	**0.0011 (0.94–0.850)**	0.730	0.1178 **(0.554–0.964)**	0.821	**0.0159 (0.721–0.935)**
Midlands vs. Pee Dee	0.763	**<0.0001 (0.728–0.799)**	0.763	**<0.0001 (0.724–0.803)**	0.762	**0.0376 (0.624–0.930)**	0.674	0.1377 **(0.470–0.966)**	0.783	**0.0028 (0.682–0.898)**
Midlands vs. Upstate	0.814	**<0.0001 (0.779–0.850)**	0.847	**<0.0001 (0.807–0.890)**	0.642	**<0.0001 (0.537–0.768)**	0.450	**<0.0001 (0.329–0.615)**	0.782	**0.0009 (0.689–0.889)**
Pee Dee vs. Upstate	1.066	**0.0225 (1.020–1.114)**	1.111	**<0.0002 (1.058–1.167)**	0.843	0.2381 (0.705–1.008)	0.668	**0.0419 (0.495–0.902)**	0.999	1.0000 (0.879–1.136)

*Bolded *p-*values and 95% CIs are statistically significant at *p* < 0.05.

### ADRD-specific incidence by PHRs

Compared to Pee Dee, the Low Country had a significantly lower AD incidence (*p* < 0.0001), Low Country also showed significantly higher incidence of Vad and Mixed dementia compared to the Midlands and Upstate regions. The Midlands also showed significantly lower AD incidence than Pee Dee (15% lower in the Midlands compared to Pee Dee; *p* < 0.0001). Additionally, statistically significant differences in incidence were observed for Vascular (*p* = 0.0376), and other types of dementia (*p* = 0.0028) for the Midlands compared to Pee Dee (Table 2).

AD incidence was significantly higher in the Pee Dee compared to the Upstate (11% higher than Upstate; *p* < 0.0002). For Mixed dementia, incidence was significantly lower in the Pee Dee compared to Upstate (33% lower than Upstate; *p* = 0.0419; Table 2).

### Age-specific incidence

Among registrants under 65 years, AD, mixed and other dementia were highest in the Upstate PHR, vascular dementia incidence were highest in the Pee Dee PHR. The Low Country recorded the lowest incidence of AD and vascular dementia, whereas the Midlands and the lowest incidences of mixed dementia and other dementias (Table 3).

**Table 3:** Incidence (per 100,000 population) of ADRD specific diagnosis by PHR and age-group in the SCADR.

PHR	Age group	Population at risk	ADRD Specific incidence			
			AD	VaD	Mixed	Other
Low Country	<65	235,854	44.095	9.752	2.120	14.840
	65–74	140,974	289.415	36.886	11.350	47.526
	75–84	61,438	1634.168	99.287	50.457	224.617
	85 and above	15,043	7179.419	252.609	159.543	737.885
Midlands	<65	253,428	48.929	11.443	1.973	11.443
	65–74	150,186	270.718	32.775	7.866	52.439
	75–84	64,489	1637.489	86.837	27.912	238.800
	85 and above	16,568	6458.233	265.572	96.572	676.002
Upstate	<65	205,352	80.837	15.583	3.896	25.809
	65–74	160,249	321.999	54.915	16.849	76.132
	75–84	71,864	1842.369	154.458	69.576	278.303
	85 and above	18,673	7229.690	396.294	198.147	797.944
Pee Dee	<65	194,151	69.534	19.057	3.090	14.937
	65–74	130,608	388.184	37.517	13.782	78.862
	75–84	53,351	1913.741	110.588	31.864	309.273
	85 and above	11,942	7955.116	326.578	117.233	695.026

Among registrants aged 65–75 years, the Pee Dee PHR recorded higher incidence rates of AD and other dementia types, while incidences of vascular and mixed dementias were highest in the Upstate. The Midlands showed the lowest incidence of AD, vascular, and mixed dementias, whereas the Low Country had the lowest incidence of other dementia types (Table 3).

In the 75–84 age group, the Pee Dee PHR reported the highest incidence rates of AD and other types of dementia, while vascular and mixed dementias were higher in the Upstate PHR. The Midlands consistently had the lowest incidence across all ADRD types (Table 3).

Among registrants aged 85 and older, AD incidence was higher in the Pee Dee, whereas vascular, mixed and other dementia type were higher in the Upstate. The Midlands reported the lowest incidence rates for AD, mixed and other dementia type (Table 3).

### Sensitivity analyses

Missing data were associated with age-group (*p* < 0.0001) and ADRD specific diagnosis (*p* < 0.0001) with small to moderate effect sizes (Cramer’s V = 0.15 and 0.12, respectively; Supplementary Table 1). Missingness was weakly associated with sex (*p* = 0.020, Cramer’s V = 0.018). These findings suggest that missingness is not completely at random but may be missing at random on age and ADRD specific diagnosis.

Sensitivity analyses were conducted to assess whether excluding the missing zip codes or counties in the analysis may influence the estimation of the true regional incidence rates. Using the proportional redistribution method, we compared full sample incidence estimates to estimates based on the complete case analysis (19, 20). The results showed a consistent increase of about 20.6% across all PHRs (complete case analyses underestimates the true incidence). Additionally, to assess the robustness of PHR estimates under extreme assumptions (21, 22), we assigned all cases with missing zip codes to the region with the lowest observed incidence (Midlands) and the highest (Pee Dee). The incidence rates increased accordingly in the Midlands and Pee Dee while the other PHRs remained stable (Supplementary Figure 1).

## Discussion

This study is the first to characterize the incidence of ADRD by PHRs in South Carolina using the SCADR data. Our study showed that the crude incidence of ADRD differed significantly across the PHRs. The incidence of AD was higher in the Pee Dee compared to the other three PHRs. The incidence of VaD, Mixed dementia, and other types of dementia was higher in the Upstate PHR compared to the other PHRs. We observed a statistically significant difference in ADRD-specific incidence across all PHRs.

Our findings of higher incidence of ADRD in the Pee Dee Region are consistent with previous studies indicating the elevated risk among individuals in socioeconomically disadvantaged regions in the Southeast of the US including South Carolina (23, 24). Similarly, a study in New Zealand reported about 43% risk of developing dementia among individuals residing in socioeconomically disadvantaged neighborhoods (25).

The high incidence of ADRD in the Pee Dee PHR may be attributed to many factors including rurality. According to the Office of Management and Budget (OMB) (26), definition of an Urban vs. Rural County, the Pee Dee PHR is predominantly rural. Research shows that dementia risk factors, are influenced by environmental factors such as living in areas with high mortality rates, including rural settings (27). Additionally, data suggest that in the US, the incidence of dementia among older adults 65 years and older was higher among people with less than a high school education and people below the federal poverty line (3). The Pee Dee PHR has the highest vulnerability index in South Carolina, indicating inequitable access to healthcare and essential resources (10). Previous research shows the association between poverty and the risk of ADRD diagnosis (28). A systematic review to assess the association between neighborhood characteristics and cognition reported evidence of an association between socioeconomic status such as poverty, and cognition (28). Rural counties are more likely to be socioeconomically disadvantaged with increased risk factors for ADRD such as increased toxicants, and poor health outcomes due to limited healthcare access (29). The risk of cognitive impairment is more pronounced in people living in deprived areas (30).

The role of education in the diagnosis of ADRD cannot be overemphasized. Higher education (at least 16 years) increases cognitive reserve, which has been reported to be associated with less evidence of biomarkers of Alzheimer’s disease (31). Higher education influences a person’s lifestyle choices including diet, physical activity, and other decisions through lifelong learning which may delay the onset of ADRD. In rural counties, there are limited resources, including limited access to education. The higher incidence of ADRD in Pee Dee therefore may be suggestive of the cascading effect of limited resources including education. The persistent teacher shortages in the Pee Dee compared to the other public health PHRs (32) may indicate broader educational disparities, limiting access to quality education, and a continuous cycle of lower socioeconomic status that ultimately influences public health outcomes, including ADRD incidence.

It has also been suggested that the higher incidence of ADRD diagnoses in rural counties may be attributed to the higher prevalence of chronic conditions and morbidities which are precursors to ADRD diagnosis (33). Cardiovascular disease risk factors including hyperlipidemia, diabetes, hypertension and physical inactivity have been shown to be associated with dementia and dementia severity, progressing to Alzheimer’s disease (7, 34, 35). South Carolina is in Southeastern US with higher stroke incidence and mortality, often described as the stroke belt (36). Thus, the prevalence of cardiovascular disease risk factors is high in South Carolina. However, data shows disparity in the occurrence of these factors. Pee Dee PHR leads the regions in the prevalence of obesity, diabetes, stroke, hypertension and physical inactivity (37).

Our findings were consistent with previous studies that found an association between rural counties (areas) and higher ADRD incidence (38–40).

We observed a higher incidence of AD, VaD, and Mixed dementia among participants younger than 65 years in the Pee Dee PHR compared to the other PHRs. Although we can only speculate reasons for this observation, lower educational attainment and access to healthcare resources as described previously may best explain this observation. Previous studies have shown that an increase in educational attainment was associated with a reduction in the incidence of ADRD. In a study to describe the temporal trends in the incidence of dementia among participants in the Framingham study, incidence declined by 44% in the third epoch, observed among only participants with at least a high school diploma. The mean age of diagnosis/incidence increased from 80 years in the first epoch to 85 years during the fourth epoch (41).

Research shows that increased physical activity in younger ages decreases the risk of developing AD, VaD (42). Exercise increases blood flow to the cerebral part of the brain and decreases insula perfusion. The insula is affected in the early stages of AD, with hyper perfusion in the insula commonly reported among persons at risk of AD (43, 44). The Pee Dee region has the highest prevalence of physical inactivity in South Carolina (37). There is evidence of significant clusters of physical inactivity and obesity in rural counties compared to urban counties in South Carolina (45). People living in rural areas have less access to traditional or formal recreational parks, and increased crime in these areas discourage outdoor exercises compared to safe sidewalks, modern fitness centers and playgrounds in urban settings (46–48). Our findings were consistent with Rahman et al. (40), who reported that age at ADRD diagnosis/incidence was higher among younger populations in rural and micropolitan counties compared to metropolitan counties.

The incidence of AD was consistently higher among participants aged 65–75 years, 75–84 years and 85 years and above in the Pee Dee compared to other PHRs.

The incidence of VaD was consistently higher in the Upstate PHR among participants aged 65 years and above compared to the other PHRs. Decreased blood supply to the brain due to blocked or ruptured vessels in the brain or complications of hypertension increase the risk for VaD (49). Risk factors associated with cardiovascular health including smoking, diabetes, and hypertension are also associated with an increased risk of VaD (50–52). South Carolina is among the top 10 states with a high incidence of cardiovascular diseases in America, additionally, Greenville County, one of the most populous counties in South Carolina and in the Upstate PHR ranks in the top third in the state for heart disease (53). Vascular risk factors increase the risk of stroke, and a history of stroke also increases the risk of VaD (41), therefore, we cannot rule out the possibility of this being the reason for the observed higher incidence of VaD in the Upstate PHR. According to the CDC Wonder Data, Oconee County in the Upstate PHR is a noted hotspot for high cholesterol among adults 18 years and above (37). Hyperlipidemia is vascular risk factor associated with CVD and dementia, because of its role in atherosclerosis and cognitive impairment due to cerebral hypoperfusion or embolism (54). The Upstate PHR has the high cluster of healthcare facilities in South Carolina (45). A high cluster of healthcare facilities provide opportunities for hospital to improve performance through improved surveillance and integrated healthcare services. Therefore, more cases of VaD may be diagnosed more in the Upstate PHR compared to the others.

Previous studies have reported a strong association between cognitive health and healthcare access (55). The availability of physicians or primary care providers plays a key role including the management of risk factors, identifying patient cognitive changes overtime and timely diagnosis. According to the *Lancet commission on Dementia Prevention, intervention, and Care*, improving access to primary healthcare has the potential to delay or even prevent 40% of dementia cases (56).

Population risk differences across geographic locations, population educational level, and preexisting health conditions including cardiovascular disease, the availability of healthcare providers including neurological specialties, and the knowledge and care-seeking behaviors may account for these variations (56).

### Strengths and limitations

We used data from the SCADR, that are relatively large and representative of the population (in comparison to CDC and the National Alzheimer’s Association report/data). Including the natural logarithm of the population at risk as an offset variable in our model allowed us to account for rates and to be able to compare the different population sizes in the PHRs. This is a major strength of our study. Additionally, by conducting sensitivity analyses, we demonstrated the robustness of our PHR estimates under different assumptions regarding missing data, which strengthen confidence in the validity of our findings.

Our study had some limitations; we did not assess for vascular factors in the PHRs as well as other detailed risk factors including education, lifestyle factors (smoking, alcohol use, environmental pollution, sleep pattern), and therefore we were not able to determine their association with the incidence of ADRD in each PHR. Due to the nature of registry data we cannot rule out the possibility of underreporting of data, underdiagnoses in the PHRs or missing county or zip codes or measurement errors; these have the potential to introduce bias. Of important note, the study was conducted after the COVID-19 lockdown period in South Carolina, while we do not have evidence to suggest this may have affected reporting of ADRD cases, there is a potential. Additionally, this study is a retrospective cohort analysis based on individuals diagnosed and reported in the SCADR; as such, the incidence estimates may not fully capture the true burden of ADRD in the entire South Carolina population. The use of the registry data limits our ability to control for all potential confounders, therefore we cannot rule out potential residual confounding.

## Conclusion

The incidence of ADRD differs significantly across the PHRs, with the Pee Dee PHR having higher ADRD incidence. The incidence of AD, Mixed and other dementia types among participants younger than 65 years was higher in the Upstate PHR, and consistently higher incidence among participants 65 years and older in the Pee Dee PHR.

Additionally, the incidence of VaD was consistently higher among participants 65 years and older and in the Upstate PHR. Data from the Registry highlights significant differences in ADRD incidence in the PHRs, this suggests possible disparities in healthcare access, socioeconomic conditions, and rural/urban factors.

Comparing the incidence in the PHRs provides a unique opportunity to identify regional patterns and disparities for targeted interventions and increase the availability of resources regionally. Efforts to enhance early screening among younger populations in the Pee Dee region should be prioritized. Future research should delve deeper into these disparities within the younger populations in the PHRs. Findings in this research should be interpreted with caution as this is an observational study and is not intended to establish causal associations.

## Data Availability

The datasets presented in this article are not readily available because the data used for this study is subject to licenses and restrictions. Data request and access can be obtained at osa-sc.org/programs/alzheimers-disease-registry. Requests to access the datasets should be directed to Maggi Miller, CHANDLMJ@email.sc.edu, https://osa-sc.org/programs/alzheimers-disease-registry.
