# Supplementary material for: Regional differences in the incidence of Alzheimer’s disease and related dementias in South Carolina
Source: Front Neurol. 2025 Sep 5;16:1584127. doi: 10.3389/fneur.2025.1584127 (PMC12447272; doi:10.3389/fneur.2025.1584127)
Supplement: Supplementary file 1 [file Table_1.docx]

**Supplementary tables and figures**

Supplementary table 1: Assessing the association between missing data and demographic variables

| **Variable** | **DF** | **Chi-Square** | **P value** | **Cramer’s V** |
| --- | --- | --- | --- | --- |
| Age Group | 3 | 406.059 | <.0001 | 0.1515 |
| Sex | 1 | 5.4123 | 0.0200 | 0.0175 |
| ADRD subtype | 3 | 253.649 | <.0001 | 0.1157 |
| Race | 2 | 8414.996 | <.0001 | 0.6663 |

**Supplementary table 2: Proportional Redistribution Sensitivity Analysis**

| **PHR** | **Full Sample** | **Complete Cases** | **% Difference** |
| --- | --- | --- | --- |
| Upstate | 1058.77 | 840.31 | 20.67 |
| Midlands | 861.31 | 683.63 | 20.62 |
| Low Country | 962.63 | 763.99 | 20.63 |
| Pee Dee | 1128.74 | 895.89 | 20.65 |

**Supplementary figure 1**


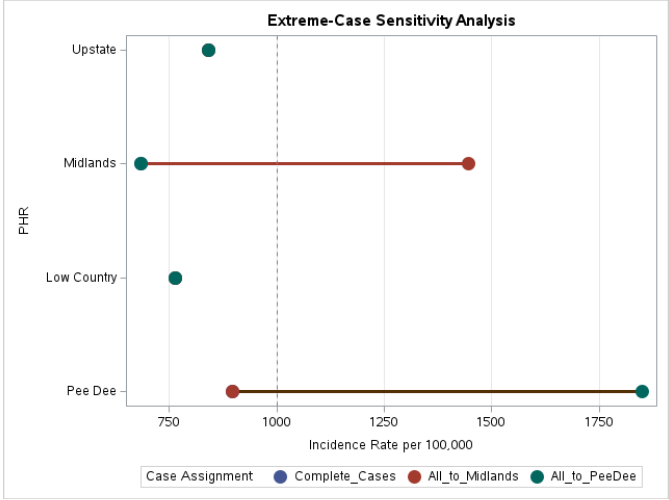


**Supplementary figure 1. Extreme-Case sensitivity Analysis of ADRD incidence by PHRs in South Carolina.**
